# The discovery and characterization of novel soluble guanylate cyclase stimulators

**DOI:** 10.1186/2050-6511-16-S1-A5

**Published:** 2015-09-02

**Authors:** Jaime L Masferrer

**Affiliations:** 1 Ironwood Pharmaceuticals Inc, Cambridge, MA, USA

## 

Soluble guanylate cyclase (sGC) is an endogenous receptor for nitric oxide (NO). Binding of NO to sGC activates the enzymatic activity, catalyzing the conversion of GTP into cGMP. cGMP acting as a second messenger, evokes a number of physiological responses including the regulation of blood pressure by relaxing vascular smooth muscle cells. Elevated reactive oxygen species can reduce the bioavailability of NO leading to dysregulation of the NO-cGMP signaling pathway and this has been associated with multiple diseases including diabetes, hypertension, and heart failure. Stimulators of sGC synergize with NO to enhance NO signaling and thus represent a new therapeutic mechanism. The sGC stimulator riociguat (Bayer) was recently approved for the treatment of pulmonary arterial hypertension and inoperable chronic thromboembolic pulmonary hypertension. We have discovered a novel pyrazole-pyrimidine class of sGC stimulators with drug-like properties. These compounds synergize with NO to stimulate sGC in isolated enzyme and whole cell assays, relax pre-constricted aortic smooth muscle preparations ex vivo, and potently reduce blood pressure in normotensive and spontaneously hypertensive rats (SHR). Additionally, sustained and dose-dependent blood pressure lowering effects were observed in the Dahl salt-sensitive rat model of hypertension and heart failure. Furthermore, sGC stimulator treatment resulted in protection from end organ damage and reduction in biomarkers of fibrosis and inflammation in rats. The blood pressure lowering effects observed in the rat with a sGC stimulator can be combined with existing antihypertensive mechanisms underscoring the potential utility of this mechanism for the treatment of resistant hypertension. We observed differences between various sGC stimulators in their pharmacological and pharmacodynamic effects, which may enable the therapeutic use of these molecules in a variety of different indications.

